# Psychometric Properties of Sensory Processing and Self-Regulation Checklist (SPSRC)

**DOI:** 10.1155/2019/8796042

**Published:** 2019-12-01

**Authors:** Cynthia Y. Y. Lai, Trevor W. K. Yung, Ivan N. B. Gomez, Andrew M. H. Siu

**Affiliations:** Department of Rehabilitation Sciences, The Hong Kong Polytechnic University, Hong Kong

## Abstract

**Background:**

Some children may encounter difficulties in processing sensory stimuli, which may affect their ability to participate in activities of daily living. Self-regulation abilities may also affect children on how to process different sensory experiences. The Sensory Processing and Self-Regulation Checklist (SPSRC) was developed as a single, parent-reported instrument for the examination of sensory processing and self-regulation difficulties in children.

**Aims:**

This study is aimed at evaluating the psychometric properties of the SPSRC and examine the patterns of self-regulation and sensory processing in children with and without autism spectrum disorder (ASD).

**Methods and Procedures:**

The contents of the SPSRC were validated by a group of experts, and a field test was subsequently conducted to examine the reliability and validity of this instrument in a sample of 997 typically developing children and 78 children with ASD.

**Outcomes and Results:**

The results of the validation and field test analyses suggest that the SPSRC exhibits high internal consistency, good intrarater reliability, and a valid ability to measure and discriminate sensory processing and self-regulation in children aged 3–8 years with and without ASD.

**Conclusions and Implications:**

The current results supported the reliability and validity of SPSRC to assess a child's sensory processing and self-regulation performance in activities of daily living. The study findings warrant further investigation to compare the performance of the SPSRC with laboratory-based tests, as this would better elucidate sensory responsivity in children with sensory modulation disorders from both clinical and research perspectives.

## 1. Introduction

Sensory processing refers to the manner by which the central and peripheral nervous systems process external sensory stimuli [1] and involves the ability to register and manipulate information and integrate different types of received sensations [2]. However, some children may encounter difficulties in processing these sensory stimuli, which may affect their abilities to participate in activities of daily living. The estimated incidence rates of sensory processing difficulties range from 40% to 80% among children with disabilities and from 5% to 16.5% among their typically developing counterparts ([2–4]. “Sensory processing disorder” is not a medical classification, but rather a nosologic classification proposed by Miller et al. [5] to describe the presence of several sensory behavior symptoms and the resulting effects on a child's abilities to function and participate in activities [6]. Sensory modulation disorder, which is commonly observed in children with autism spectrum disorder (ASD), represents one sensory processing disorder pattern.

### 1.1. Definition of Sensory Modulation Disorder

Sensory modulation disorder is one of three sensory processing disorder patterns. The rationale of sensory processing disorder is based on sensory integration theory as put forth by Ayres [7], wherein varying degrees of unusual sensory responses reflect underlying neurophysiological deficits. Three subtypes of sensory modulation disorder have been identified: sensory overresponsivity, sensory underresponsivity, and sensory seeking or craving. Children with sensory overresponsivity react more quickly, more intensely, or for longer than typical durations, whereas those with sensory underresponsivity disregard or fail to respond to environmental sensory stimuli and do not appear to detect incoming sensory information. Children exhibiting sensory-seeking behavior tend to crave unusual amounts or types of sensory input and appear to have an insatiable desire for sensation.

### 1.2. The Needs to Understand Self-Regulation Mechanism in the Sensory Modulation Disorder of People with ASD

Studies have shown that most people with ASD exhibit sensory overresponsivity, underresponsivity, or seeking behavior [2, 8–12]. Previous understandings of these types of behavior have largely focused on the behavioral approach (stimulus-response pattern), wherein patterns of behavioral responses have been classified as overarousal, underarousal or fluctuating arousal levels, deficits in multisensory integration, or abnormalities in sensory integration [3, 13–17]. Building on Ayres' sensory integration theory, Dunn [18] further proposed a sensory processing model that explained the response pattern (i.e., overresponsive, underresponsive, or sensory-seeking behavior) as a combination of the neurophysiological threshold and self-regulation strategies. In other words, approaching or avoiding the stimulus is considered a self-regulatory behavioral strategy. However, an understanding of the neurophysiological mechanism that underlies these behavioral strategies is essential.

Recent studies have found that children with ASD exhibit reduced parasympathetic functioning at rest compared with their normal peers. Children with ASD have also been found to exhibit an unusual facilitation or suppression of parasympathetic or sympathetic activity upon processing sensory information [19–24]. Autonomic activity is an important mediator of responses in stressful or challenging situations [25–29]. From a physiological perspective, suboptimal autonomic functioning may contribute to the maladaptive responses of children with ASD to daily life scenarios. Therefore, it may be useful to simultaneously evaluate self-regulation behaviors and sensory information processing during activities of daily living.

### 1.3. Self-Regulation during Daily Sensory Events

An individual must be able to adapt and respond appropriately to successfully interact with the environment. The process by which the self adapts to the internal and external environment is called self-regulation [30]. To achieve this goal, fundamental self-regulation capacities (e.g., physiological, cognitive, and psychosocial functioning) are required to support behavioral regulation. Kopp [31] proposed five phases in the hierarchical development of self-regulation in children: neurophysiological modulation, sensorimotor modulation, control, self-control, and self-regulation. However, most children with ASD were found to exhibit impaired fundamental capacities for self-regulation, which hinders their ability to regulate their behavior in response to daily events. In fact, researchers previously suggested that the behavioral and physiological regulation of responses to sensory stimuli were related in children with ASD [32, 33]. In other words, self-regulation and sensory processing issues typically coexist among children with ASD. Therefore, it is important to discern any possible contributor such as self-regulation to individual behavioral patterns in response to sensory events.

### 1.4. Developing a Tool to Measure Both Self-Regulation Abilities and Sensory Processing Abilities

Standardized tests and checklists/questionnaires are currently used to identify children with sensory modulation disorders. Checklists or questionnaires are commonly used in clinical settings to obtain valuable information regarding the child's performance in home or school environments ([34–36]. These checklists are useful for quantifying the occurrence of behaviors in response to various types of sensory stimuli. Moreover, an understanding of a child's fundamental capacity for self-regulation (e.g., physiological, cognitive, and psychosocial functioning) may clarify the problem underlying the behavioral patterns displayed during sensory events. The currently available checklists used to ascertain sensory modulation in children primarily assess behavioral responses. Gomez et al. [37] conducted a systematic review of the behavioral and physiological methods used to research the regulation of sensory responses and concluded that while the currently used checklists/questionnaires were useful for describing behavioral responses to sensory events, they failed to provide insights into the underlying neurophysiological mechanisms supporting self-regulation. For instance, although the Chinese Sensory Profile (CSP; [38]) tool is available for the Chinese population, other information about the fundamental capacities of children to self-regulate is lacking. Therefore, a measure that incorporates aspects of self-regulation from a neuropsychophysiological perspective is needed to clarify how children regulate sensory responses.

At the time of this research, limited evidence was available regarding the use of data from a single checklist to provide information about children's sensory processing and self-regulation behaviors from a combined neuropsychophysiological perspective. As mentioned earlier, children with ASD commonly exhibit sensory processing and self-regulation symptoms. Therefore, a Sensory Processing and Self-Regulation Checklist (SPSRC; [39]) was developed to examine sensory processing and self-regulation in children aged 3–8 years. The SPSRC is based upon Ayres' sensory integration theory, in addition to theories of information processing and self-regulation, which consider the interaction of the regulation and processing of sensory information from the external environment in the body. Unusual sensory behavioral response patterns (e.g., overresponsivity, underresponsivity, or sensory-seeking behavior) may be attributed to sensory processing deficits, which are related to self-regulatory issues supported by neuropsychophysiological functions. However, it remains uncertain if these sensory behavioral response patterns (e.g., underresponsivity versus sensory-seeking behavior) are attributable to the same latent factor.

The SPSRC was developed to provide a single instrument for the evaluation of sensory processing and self-regulation difficulties in children. The SPSRC comprises two sections: self-regulation ability and sensory processing ability (see appendix). The self-regulation ability section aims to identify children's difficulties with behavioral regulation and comprises items that reflect the ability to self-regulate. The sensory processing ability section aims to identify behavioral patterns in response to sensory input and to quantify the extent of difficulties encountered by children upon receiving different types of sensory input. This section includes items that reflect the behavioral response patterns of children upon receiving different kinds of sensory input. The SPSRC items are written in Chinese and reflect the culture and environment of Hong Kong. The information obtained from the SPSRC may supplement the current understanding of sensory processing difficulties in children.

This study is aimed at evaluating the psychometric properties of the SPSRC and to examine the patterns of self-regulation and sensory processing in children with ASD. Children's fundamental physiological, cognitive, and psychosocial abilities could contribute to behavioral regulation in the context of sensory processing. We hypothesized that the SPSRC was a valid tool for the identification of sensory modulation disorder and that the SPSRC scores of children with ASD would be lower than those of their normal peers.

## 2. Methods

### 2.1. Participants

Two groups of Chinese children aged 3–8 years were recruited for this study. The two groups included typically developing children and children with ASD.

#### 2.1.1. Typically Developing Children

To obtain a representative sample, participants were recruited using multistage cluster sampling [40]. The population was divided first by geographical region and then by age. Service units, including kindergartens, kindergarten-cum-child-care centers, nurseries, and mainstream primary schools, were randomly selected by district from the database provided by the Education Bureau and Social Welfare Department of the Hong Kong Special Administrative Region (HKSAR) government and invited to participate in this study. Thirty service units across different regions of Hong Kong agreed to participate, and appropriately aged children at each unit were invited to enroll. Children with any formally diagnosed developmental disabilities were excluded. Overall, 1,039 children were recruited from the participating units, and 42 were excluded from the analysis due to missing personal data. This sample of 997 typically developing children (52.5% male, 46.9% female, 0.6% with missing sex data) aged 37–107 months formed and consisted the normative group.

#### 2.1.2. ASD Group

A convenience sample of 78 children with ASD (79.5% male, 20.5% female) aged 43–106 months was recruited from 22 special child-care centers, early education and training centers, kindergartens, kindergarten-cum-child-care centers, nurseries, and mainstream primary schools. The children were diagnosed with autism, autistic disorder, Asperger syndrome, or ASD by a pediatrician affiliated with the Child Assessment Service in Hong Kong.

### 2.2. Instrumentation

#### 2.2.1. SPSRC

As noted above, the SPSRC comprises two sections. Part 1, which tests self-regulation ability, comprises three scales: (1) physiological, (2) social/cognitive/emotional, and (3) facing changes or challenges. Part 2, which tests sensory processing ability, comprises six scales: (1) auditory, (2) visual, (3) tactile, (4) gustatory/olfactory, (5) vestibular, and (6) proprioceptive. Parents were required to report their child's typical condition during the previous 3 months on a 5-point Likert scale (never = 5, seldom = 4, sometimes = 3, most of the time = 2, always = 1; opposite polarity in some items). A lower score corresponded to a less favorable performance.

#### 2.2.2. The Chinese Sensory Profile (CSP) [38]

The CSP is a validated version of Tseng's [41] Chinese Sensory Profile which was original from The Sensory Profile [34] and was reduced to 100 items in order to enhance the cultural relevance [41]. It includes 6 sensory system scales (auditory processing, visual processing, taste/smell processing, body position, movement, and touch processing) and 2 behavioral category subscales (activity level and social/emotional responses). Each item was rated by parents in a 5-point Likert scale ranging from 1 (always or 100%) to 5 (never or 0%).

### 2.3. Procedure

To recruit participants, verbal or written consent was first obtained from the heads of the service units or school principals. These individuals had each received a study information package that included an invitation letter to the service unit or school, an information sheet about the purpose and procedure of the study, an invitation letter for parents, a participant demographic questionnaire, and a consent form. Informed consent was obtained from the children's parents. After receiving the completed demographic questionnaire, the parents of the typically developing children and children with ASD were asked to complete the SPSRC. Some parents of typically developing children completed both the SPSRC and the CSP to evaluate convergent validity. Additionally, some parents of typically developing children completed the SPSRC twice (at a 2-week interval) to evaluate intrarater reliability. All these typically developing participants were randomly selected from 997 typically developing participants after matching their age and sex with ASD participants.

### 2.4. Data Analysis

The psychometric properties of the SPSRC were examined. Reliability was established by performing internal consistency and interreliability testing. The internal consistency of the SPSRC was examined using separate Cronbach's *α* analyses of Part 1, Part 2, and the composite SPSRC scores. The intrarater reliability was determined by examining the intraclass correlation coefficient between the two ratings given by parents who completed the SPSRC twice. Cronbach's *α* was used as a coefficient of reliability, and a threshold of >0.70 was set to indicate acceptable-to-excellent internal consistency [42]. The validity of the tool was assessed through tests of the factor analysis and convergent and divergent validity. A principal component analysis was used for the factor analysis, and a factor loading threshold of >0.30 was set to indicate practical significance (i.e., that the item/s are influenced by the underlying construct of the tested factor) [42]. Convergent validity was examined using the Pearson correlation coefficient between the SPSRC and Chinese Sensory Profile. For discriminant validity, the SPSRC scores of typically developing children and children with ASD were compared through a multivariate analysis of variance. SPSS 20.0 (SPSS, Inc., Chicago, IL, USA) was used for all statistical analyses, and the significance level was set at a critical level of 0.05.

## 3. Results

### 3.1. Development of the SPSRC

Several experts in sensory processing disorders and developmental disabilities from hospitals, child assessment centers, early education and training centers, and special child-care centers were recruited to review the first draft of the SPSRC. The panel members were asked to complete a questionnaire to rate the relevance and representativeness of the items to the particular constructs of sensory processing ability and self-regulation ability using a 5-point Likert scale (strongly disagree = 1, disagree = 2, agree = 3, strongly agree = 4, totally agree = 5). The panel members also met to discuss and review their findings. After the initial draft was amended, all group members rated all of the items on the second draft of the SPSRC “strongly agree” or “totally agree.” This version, which contained 132 items, was applied during the field test.

### 3.2. Internal Consistency

The consistency of responses to the items of the SPSRC was tested to determine whether each of the subscale and composite scores measured the same general construct. Cronbach's *α* coefficients were 0.89 and 0.97 for the items in Part 1 (37 checklist items) and Part 2 (93 checklist items) of the SPSRC, respectively. Overall Cronbach's *α* coefficient for the SPSRC composite (130 checklist items) was 0.97.

### 3.3. Intrarater Reliability

Intrarater reliability was tested among a sample of 36 typically developing children aged 3–8 years, of whom 21 were male and 14 were female. (Sex data were missing for one subject.) The parents of these 36 children completed the SPSRC twice (at a 2-week interval) to examine the consistency between the ratings provided by the same rater. The intraclass correlation coefficients for Part 1 (37 items), Part 2 (93 items), and the SPSRC as a whole (130 items) were 0.91, 0.95, and 0.94, respectively.

### 3.4. Factor Analysis

The latent factor of the SPSRC was examined via exploratory factor analysis. The scree plots suggested a two- to four-factor solution for the interpretation of Part 1 and a two- to five-factor solution for Part 2.

For Part 1 of the SPSRC, the promax rotation method yielded a four-factor solution with a principal component analysis. In the four-factor solution, components 1–4 accounted for 38.9% of the accumulated variance. The two items with the lowest factor loading were removed from the second draft of the SPSRC. The final version of Part 1 of the SPSRC has 37 items. Factor 1, “emotional regulation–facing challenges,” comprises 10 items (factor loading range 0.56–0.83) and reflects a child's emotional regulation abilities when facing challenging situations. Factor 2, “emotional regulation–facing changes,” comprises 12 items (factor loading range 0.45–0.77) and reflects a child's emotional regulation abilities when facing changes in routines or events. Factor 3, “physiological regularity and response to soothing,” comprises nine items (factor loading range 0.44–0.65) and reflects a child's physiological patterns (e.g., sleeping, bladder and bowel and eating patterns) and responses to soothing by adults. Factor 4, “autonomic activity,” comprises six items (factor loading range 0.32–0.55) and reflects the child's autonomic responses (e.g., palm sweating). Because two items (“seems drowsy even 30 minutes after getting up from bed” and “enjoys sucking or putting things into mouth”) on the second draft could not be strongly justified under any factor, they were removed. Therefore, the finalized version of the SPSRC comprised 37 items. The internal consistencies of factors 1, 2, 3, and 4 were 0.89, 0.85, 0.72, and 0.50, respectively.

For Part 2 of the SPSRC, the quartimax rotation method yielded a four-factor solution with a principal component analysis. In the four-factor solution, components 1–4 accounted for 42.6% of the accumulated variance. With reference to the factor loadings of all items from all four factors, no items were removed from the second draft of the SPSRC. Factor 1, “sensory-seeking behavior,” comprises 35 items (factor loading range 0.35–0.75) and reflects a child's tendency to demonstrate sensory-seeking behavior. Factor 2, “sensory underresponsivity,” comprises 23 items (factor loading range 0.35–0.67) and reflects a child's diminished or lack of response to sensory stimulation. Factor 3, “sensory overresponsivity,” comprises 29 items (factor loading range 0.33–0.68) and reflects a child's exaggerated response toward sensory stimulation. Factor 4, “stability of sensory responsivity,” comprises six items (factor loading range 0.23–0.50) and reflects a child's stability in response to sensory stimulation across situations. As all of the items in the second draft were retained, the finalized version of the SPSRC contained 93 items. The internal consistencies of factors 1–4 ranged from 0.93 to 0.95.

### 3.5. Convergent Validity

The construct of the SPSRC was further evaluated using the multitrait-multimethod matrix. The sample for convergent validity comprised 82 typically developing children aged 3–6 years (46 boys and 36 girls). All parents completed both the SPSRC and CSP within 2 weeks. The Pearson correlation coefficient between the total scores of the two checklists was 0.931 (*p* < 0.001). The correlations between the scores of the sensory domains of the SPSRC (Part 2) and the CSP ranged from 0.57 to 0.84 (Table 1). The correlations between the scores of some of the similar latent factors of the SPSRC and the CSP ranged from 0.55 to 0.82 (Table 2).

### 3.6. Discriminant Validity

Data from an age- and sex-matched sample of 78 children with ASD and 78 typically developing children aged 3–8 years were subjected to a multivariate analysis of variance. These 78 typically developing participants were randomly selected from the 997 typically developing children after matching their age and sex with those children with ASD. Typically developing children received significantly higher scores on all of the scales, compared with children with ASD (see Table 3).

## 4. Discussion and Conclusions

The different evaluation methods (e.g., internal consistency, intrarater reliability factor analysis, convergent validity, and discriminant validity) used in this study revealed the good psychometric properties of the SPSRC. As hypothesized, the factor analysis of Part 2 of the SPSRC identified three patterns of sensory modulation disorder: sensory overresponsivity, sensory underresponsivity, and sensory-seeking behavior. The SPSRC scores of children with ASD were significantly lower than those of their normal peers.

The results reveal several issues that may require further attention from both clinicians and researchers. First, this study included only participants between 3 and 8 years of age. At this age, physiological, cognitive, social, and emotional development are not yet complete. Although physiological functioning is the basis for higher order functions, especially in young children [28], autonomic function varies with age [43–45]. Potentially, the contributions of these developing aspects to behavioral regulation may differ between young and older children. Therefore, the results of this study may not be generalizable to children older than 8 years of age.

Second, the results indicate that the SPSRC as a whole (130 items) has a high level of internal consistency. The descriptions of the items in Parts 1 and 2 are different. Those in Part 1 reflect the fundamental capacities supportive of behavioral regulation but do not describe any context related to sensory events. Part 2 includes items reflective of behavioral responses toward the sensory stimuli encountered during daily life. The SPSRC adopted Kopp's model of self-regulation development in children. The results of this study imply that items reflecting the fundamental capacities supportive of behavioral regulation in daily life (i.e., nonsensory-specific context) and behavioral responses to sensory stimuli encountered in daily life may have similar constructs. Additionally, the results suggest that clinicians and researchers should consider the extent of the fundamental capacities (e.g., behavioral regulation in nonsensory-specific daily life scenarios) that contribute to behavioral regulation when encountering daily sensory events. Nevertheless, the internal consistency of the SPSRC as a whole exceeded 0.90, which may reflect the possible redundancy of similar items. Hence, it is worthwhile to consider the elimination of some items from the SPSRC or the development of a short form.

Third, the factor analysis identified four latent factors for Part 1 (self-regulation ability): emotional regulation-facing challenges, emotional regulation-facing changes, physiological regularity, and response to soothing and autonomic activity. However, the factor loading on the items corresponding to autonomic activity was lowest among the four factors. This could be explained by the nature of the items reflective of different types of autonomic activity (e.g., “has excess palm sweating during daily activity” or “has retching, nausea or gags easily”). For Part 2 (sensory processing ability), four additional latent factors were identified: sensory-seeking behavior, sensory underresponsivity, sensory overresponsivity, and stability of sensory responsivity. Reports have suggested that the mechanism underlying sensory-seeking behavior (e.g., underarousal) is similar to that underlying sensory underresponsivity [15, 34, 46]. Our current results, however, suggest that sensory underresponsivity is distinct from sensory-seeking behavior.

Fourth, as demonstrated by the factor analysis, the factor loading of items related to the “stability of sensory responsivity” were also high on “sensory-seeking behavior” but not on “sensory overresponsivity” or “sensory underresponsivity.” The mechanisms underlying sensory-seeking behavior and sensory underresponsivity may be distinct. Each sensory subscale of the SPSRC contains one item that reflects the stability of responses to sensory events (e.g., items described as “the child responds differently to the above [auditory] events if the events occur in front of different people, at different places, or at different times of day”). Although the rationale behind “sensory-seeking behavior” is unclear, this parameter may be a means by which one can upregulate or downregulate one's physiological condition [2, 13, 47–49]. Both “sensory-seeking behavior” and “stability of sensory responsivity” may reflect children's methods of coping with external and internal demands. Children with lower “sensory-seeking behavior” and “stability of sensory responsivity” scores may be vulnerable to changes in the internal and external environments. By actively obtaining sensory input, the body may obtain pleasure from changes in the internal environment after the sensory input. Therefore, further investigation is needed to explore the effects of sensory stimulation on changes in a person's internal environment.

Fifth, children with ASD received significantly lower scores than typically developing children on all subscales of the SPSRC, including “stability of sensory responsivity.” An understanding of the stability or consistency of responses to sensory stimulation could facilitate an understanding of the effects of particular environmental features on these children. Studies have shown weak or insignificant correlations of scores in similar sensory domains on sensory checklists across home and school environments [35, 36]. This finding could be attributed to variations between raters of the same child. Unlike previous studies, in this study, the parents acted as informants and reported the stability of their children's sensory responsivity. The parents of children with ASD reported lower sensory responsivity stability, relative to their counterparts without ASD. Parents may be more sensitive to the variations of responses from their children. However, children with ASD may also have increased difficulty coping with changes in external demands. Furthermore, a sensory stimulus may contain both physical characteristics (e.g., intensity) and nonphysical characteristics (e.g., the meaning to an individual). Recent psychophysiological studies have indicated that top-down effects play a crucial role in the processing of sensory information [50, 51]. Attention processes are necessary for the perception of sensory inputs, which limits the scope of the registered information each time [52]. However, it remains uncertain whether nonphysical characteristics of the stimulus and the attention processes of children with ASD would contribute to the lower sensory responsivity stability exhibited by this population during daily sensory events. Further investigation is required.

In summary, this study of the psychometric properties of the SPSRC supported the reliability and validity for the identification of sensory modulation disorder and for the measurement of self-regulation and sensory processing abilities in children aged 3–8 years. Nevertheless, the SPSRC is a parent report rather than a test battery, and parents' perceptions of their children's performance may vary across countries and even within the same ethnic group across different countries [53]. Therefore, validation of the SPSRC in other countries is warranted. Given the ecological validity of measuring instruments, parental reports remain valuable for examining the performances of children in real-life situations. Furthermore, children's responses to sensory stimulation may vary across environments. Therefore, the use of both standardized laboratory-based tests and a sensory checklist could be used to measure sensory responsivity in children with sensory modulation disorder in both research and clinical settings.

## Figures and Tables

**Table 1:** Correlations between sensory domain scores of the SPSRC and CSP.

SPSRC (Part 2)	CSP	*r*	*p*
A. Auditory	1. Auditory processing	0.57	<0.001
B. Visual	2. Visual processing	0.70	<0.001
C. Tactile	6. Touch processing	0.72	<0.001
D. Taste and smell	3. Taste and smell processing	0.70	<0.001
E. Vestibular	5. Movement	0.83	<0.001
F. Proprioceptive	4. Body position	0.84	<0.001

**Table 2:** Correlation between the latent factors of SPSRC and CSP.

SPSRC	CSP	*r*	*p*
Part 1			
Factor 1, emotional regulation–facing challenges	Factor 1, emotional reactivity	0.55	<0.001
Factor 2, emotional regulation–facing changes	Factor 1, emotional reactivity	0.66	<0.001
Factor 3, physiological regularity and response to soothing	Not applicable		
Factor 4, autonomic activity	Not applicable		
Part 2			
Factor 1, sensory-seeking behavior	Factor 4, sensory seeking	0.82	<0.001
Factor 2, sensory underresponsivity	Factor 3, low registration	0.56	<0.001
Factor 3, sensory overresponsivity	Factor 5, sensory defensiveness	0.67	<0.001
Factor 4, stability of sensory responsivity	Not applicable		

**Table 3:** Comparison of the scale scores of SPSRC checklist categories between typically developing children and children with ASD.

Scale		ASD (*n* = 78)		Typical development (*n* = 78)		*_p_*
		Mean	SD	Mean	SD	
Ability	Part 1: self-regulation ability					
	A. Physiological	43.1	5.4	46.1	5.0	<0.001
	B. Social/cognitive/emotional	43.4	6.7	52.0	6.6	<0.001
	C. Facing changes or challenges	45.2	7.8	50.2	5.9	<0.001
	Part 2: sensory processing ability					
	A. Auditory	60.9	8.1	67.6	6.3	<0.001
	B. Visual	53.7	7.1	59.2	4.9	<0.001
	C. Tactile	76.9	9.9	85.3	8.0	<0.001
	D. Taste and smell	52.1	7.5	59.2	5.3	<0.001
	E. Vestibular	70.2	9.7	77.5	8.3	<0.001
	F. Proprioceptive	51.5	10.4	60.6	8.5	<0.001
Aspect	Part 1: self-regulation ability	131.7	16.1	148.3	13.8	<0.001
	Part 2: sensory processing ability	365.3	44.6	409.3	33.4	<0.001
Overall ability	Part 1 + Part 2: sensory processing and self-regulation abilities	497.0	57.7	557.6	39.9	<0.001
Latent factor	Part 1: self-regulation ability					
	1. Emotional regulation, facing challenges	38.7	7.1	42.7	5.7	<0.001
	2. Emotional regulation, facing changes	36.5	6.4	43.9	6.0	<0.001
	3. Physiological regularity and response to soothing	32.5	4.5	35.4	4.9	<0.001
	4. Autonomic activity	24.0	3.4	26.2	2.7	<0.001
	Part 2: sensory processing ability					
	1. Sensory-seeking behavior	127.6	20.8	146.5	17.6	<0.001
	2. Sensory underresponsivity	95.0	13.8	108.2	8.8	<0.001
	3. Sensory overresponsivity	121.2	14.4	130.8	11.7	<0.001
	4. Stability of sensory responsivity	21.5	5.0	23.9	4.8	0.002

## Data Availability

The data used to support the findings of this study may be released upon application to Dr. Cynthia Lai, who can be contacted at cynthia.yy.lai@polyu.edu.hk.

## Appendix

### SPSRC (Sample Items)

#### Part 1: Self-Regulation Ability

A. Physiological

(3) The child falls asleep easily at night (e.g., requires ≤20 minutes to fall asleep)

(5) The child experiences excessive palm sweating during daily activities

(10) The child easily experiences retching, nausea, or gagging

(11) The child's breathing is not smooth (either too rapid or too slow)

B. Social/cognitive/emotional

(5) The child becomes nervous about minor issues

(6) The child has difficulty with self-control, behaves impulsively

(10) The child is able to understand and is willing to follow adults' requests

(13) The child is able to control their desires or emotions (e.g., can pause and think before acting) so as to meet the situational demands

C. Facing transitions or challenges

(2) When playing games or doing homework, the child finds it difficult to reengage in the previous activity after being interrupted

(3) When playing games or doing homework, the child finds it difficult to shift attention from one activity to another

(7) When situated in a new environment or facing challenges, the child cries if left there or hurts himself/herself (e.g., head bumping, hand biting, and hair pulling)

(9) When situated in a new environment or facing challenges, the child hides or turns his/her face away and does not try or participate in the activity

#### Part 2: Sensory Processing Ability

A. Auditory

(4). The child does not seem aware of or respond to high-pitched sounds (e.g., whistle)

(6) When hearing a sudden sound (e.g., announcement at a railway station, air dryer), the child becomes nervous or anxious, covers ears with hands or makes complaints

(11) The child seeks auditory stimulation by making loud sounds (e.g., speaking loudly, banging toys heavily)

(15) The child responds differently to the above (auditory) events if the events occur in front of different people, at different places or at different times of day

B. Visual

(3) The child does not seem aware of or respond to obstacles in front of him/her or to water splashes on the ground

(8) The child feels uncomfortable, becomes nervous or anxious, covers the eyes with hands, or makes complaints about flashing lights (e.g., neon lights, Christmas lights)

(10) The child seeks visual stimulation by gazing at the lights for a long time

(13) The child responds differently to the above (visual) events if the events occur in front of different people, at different places, or at different times of day

C. Tactile

(4) The child does not seem aware of or does not respond to a light touch on his/her skin, hands, or legs

(8) The child becomes nervous or anxious or complains about dirt on their face (e.g., glue or paint)

(17) The child intentionally touches another's body or clothes to seek tactile stimulation

D. Gustatory/olfactory

(2) The child does not seem aware of or respond to strong odors (e.g., glue or whiteboard marker)

(6) The child is picky, makes complaints about or experiences nausea in response to a taste that is acceptable to most people (e.g., _________)

(12) The child loves to eat strongly flavored foods (e.g., very sour or salty)

E. Vestibular

(2) The child does not seem aware of or respond when he/she almost falls

(6) The child becomes nervous or anxious, refuses, or complains when he/she is lifted suddenly

(15) The child enjoys moving forward or running

F. Proprioceptive

(1) The child bumps into others, furniture, or objects unintentionally with his/her body, hands or legs when walking

(3) The child leans on the wall or furniture when standing

(7) The child loves to perform activities that require pushing and pulling

*Note*. The original items were written in Chinese. The above sample items have been translated into English to illustrate the items mentioned in this manuscript.
